# Finding a path in a methodological jungle: a qualitative research of resilience

**DOI:** 10.1080/17482631.2023.2164948

**Published:** 2023-01-05

**Authors:** Elīna Zelčāne, Anita Pipere

**Affiliations:** Department of Health Psychology and Paedagogy, Riga Stradiņš University, Riga, Latvia

**Keywords:** Critical incident technique, chronic pain, multi-method, narrative analysis, qualitative research, pluralism, resilience, thematic analysis

## Abstract

Qualitative research provides an in-depth understanding of lived experiences. However, these experiences can be hard to apprehend by using just one method of data analysis. A good example is the experience of resilience. In this paper, the authors describe the chain of the decision-making process in the research of the construct of “resilience”. s The authors justify the implications of a multi-method, pluralistic approach, and show how the triangulation of two or more qualitative methods and integration of several qualitative data analysis methods can improve a deeper understanding of the resilience among people with chronic pain. By combining the thematic analysis, narrative analysis, and critical incident technique, lived experiences can be seen from different perspectives.Therefore, the thematic analysis describes the content and answers to “what” regarding resilience, the narrative analysis describes the dynamics of resilience, and answers to “how”, while the critical incident technique clarifies the most significant experience and the answers to “why” changes happen. This integrative approach could be used in the analysis of other psychological constructs and can serve as an example of how the rigour of qualitative research could be provided.

## Introduction

Just a few decades ago, qualitative researchers put a lot of effort into discussions with quantitative researchers to prove that a qualitative research strategy can also be viewed as a scientific inquiry and can provide valid and significant knowledge. Today, qualitative research is no longer just “not quantitative research” but has developed an identity or maybe multiple identities of its own (Flick, 2018). Qualitative research is especially appropriate to study complex constructs and experiences holistically. It allows one to acquire a deeper understanding of people’s lived experiences in diverse contexts (Hong & Cross Francis, 2020) and deals primarily with an intensive rather than extensive examination of these experiences (Gough & Deatrick, 2015).

The wider use of different qualitative approaches has led to new methodological challenges. One such challenge is to support methodological integrity in keeping with a diversity of researchers’ goals and approaches (Levitt, 2021). Although qualitative research is an approach rather than a particular set of techniques, it does not mean that a researcher can choose any design or combine any methods without justification. The inconsistency between the research question and the methodology, insufficient methodological knowledge, and the lack of attention to a philosophical foundation of qualitative methodology can be mentioned as some important challenges (Khankeh et al., 2015). To overcome this challenge, a researcher must become familiar with traditional approaches and recently developed ones in qualitative research and choose the most appropriate for the given research problem and research questions.

Another challenge is how to present the findings of qualitative research in a way that they can be comprehended by both academic and non-academic readers. Therefore, the researchers need to render the qualitative research findings more “friendly” to people who may not have academic or professional backgrounds or interests, provided that the findings are still faithful (Holloway & Todres, 2007). Besides, the findings of qualitative research often make sense in a very narrow context, while outside the academic environment there is a demand for practical and more general benefits that could promote change in a wider context. Thus, researchers must provide a “thick description” of the participants and the research process, to enable the reader to assess whether these findings are transferable to their own setting (Korstjens & Moser, 2018).

Qualitative researchers often use well-trodden paths. Svend Brinkmann (2015) calls this process a “McDonaldization” of qualitative research. To cope with this trend, it is recommended to also use innovative methods to explore psychological issues in health and illness (Chamberlain & Murray, 2017) and learn from artists how to capture peoples’ attention in a more creative way (Holloway & Todres, 2007). Innovative practices in qualitative research can involve pluralisms of various kinds, creative ways of collecting and analysing data, disseminating findings, and participation in some of the ethical and practical challenges involved in qualitative research (Lamarre & Chamberlain, 2021).

Today, qualitative research is widely used in different social sciences, and psychology is one of the areas where it is expanding rapidly. The proportion of qualitative research has grown especially in the field of health psychology. One of the reasons for the current popularity of qualitative health research is the growing emphasis of policy and practitioners on patient/client experiences and practices related to prevention, illness, and use of services (Gough & Deatrick, 2015). Qualitative research design is consistent with the Chronic care model (CCM), which is a widely-used framework for organizing and providing care for people with chronic disease (Wagner et al., 2001). The CCM aims to improve the quality of care and patient outcomes by providing proactive, patient-centred, and integrated care (Spoorenberg et al., 2015). Qualitative research can provide a deeper understanding of patients’ perspectives, experiences, and treatment needs and could promote patient-centred care (O’Reilly et al., 2021; Renjith et al., 2021). When patients feel respected, are included in the decision-making process, and can express their needs and emotions without feeling judged, they report a stronger sense of alliance with the care providers (Youssef et al., 2020). Qualitative research “gives voice” to patients (Braun & Clarke, 2019; Stein & Mankowski, 2004), allowing researchers and practitioners to observe health-related issues from several perspectives and analyse qualitative data with multiple methods.

One example of such a construct that can be qualitatively studied from different points of view is the experience of resilience while living with chronic musculoskeletal pain (CMP). In this paper, we describe the chain of the decision-making process in the research of the mentioned topic, starting from the dilemma between quantitative and qualitative research strategies to the decision to combine different data analysis methods. This article focuses specifically on the discussion of how the integration of several qualitative data analysis methods can improve a deeper understanding of the formation and maintenance of resilience among people with chronic pain.

## Resilience in chronic pain: A rationale for qualitative research

The American Psychological Association defines resilience as a process of adapting well in the face of adversity, trauma, tragedy, threats, or significant sources of stress (APA, 2012). Resilience can be defined as the process of effectively negotiating, adapting to, or managing significant sources of stress or trauma. Assets and resources within the individual, their life and environment facilitate this capacity for adaptation and “bouncing back” in the face of adversity (Windle, 2011).

In previous studies, resilience has been viewed as a personality trait (Block & Kremen, 1996; Connor & Davidson, 2003, Wagnild & Young, 1993), or a dynamic process, that can lead to a positive outcome (Bonanno & Mancini, 2008; Luthar & Cicchetti, 2000; Masten, 2011; Rutter, 2006). Although there are several definitions of resilience, most of them are based on two core concepts—adversity and positive adaptation. The notion of risk and positive adaptation are fundamental to both personal characteristics and process-based conceptualizations of resilience (Vella & Pai, 2019). Some researchers use the term “adaptation” meaning both the process of adjustment and its outcome (Luthar & Cicchetti, 2000; Rutter, 2006) but recently many scholars have emphasized the three pillars of resilience—adversity, the process of adaptation, and the preservation of health functioning or positive outcome (Hiebel et al., 2021; Kunzler et al., 2018; Stainton et al., 2019).

In recent studies, researchers offer an integrative view of resilience, describing it as a multifactorial, multisystemic and context dependent construct (Miller-Graff, 2022; Sisto et al., 2019; Ungar, 2021). Individual resilience is influenced by biological, psychological, social, and ecological factors and can manifest itself in different ways, like maintaining healthy functioning despite adversity, recovering from adversity and bouncing back to homoeostasis or even bouncing forward and experiencing personal growth (Ungar, 2021).

In the context of health psychology last few years there has been a shift away from disease-focused to health-focused research (Denckla et al., 2020). Resilience is viewed not only as the absence of psychopathology but as a presence of psychological, mental, social, and spiritual capital that help to maintain the quality of life despite the illness (Babić et al., 2020). Since people with chronic pain or other chronic conditions are not able to recover fully and return to homoeostasis, resilience in this context is defined as the ability to live fulfilling life in the presence of pain (Goubert & Trompetter, 2017; Sturgeon & Zautra, 2016).Chronic diseases, especially chronic pain, can negatively affect the physical, mental, and social aspects of a person’s life. However, chronically ill people, who have higher resilience scores, tend to have less depression and anxiety. Instead, they have a better quality of life and health behaviour (Cal et al., 2015; Gheshlagh et al., 2016). The effect of resilience can manifest itself in faster recovery from the negative effects of pain, through effective preservation of positive functioning despite the presence of pain (Sturgeon & Zautra, 2010).

Although previous studies (Gonzalez et al., 2019; Hemington et al., 2017; Ramírez-Maestre et al., 2019) have confirmed that resilience plays a key role in one’s adaptation to chronic pain, several questions still need to be answered. Why some people with chronic pain are more resilient than others? What factors influenced the development of their resilience? What are people with chronic pain doing to improve and maintain their long-term resilience?

The nature of these questions has inevitably led us to the exploration of experience related to the resilience of a specific population, alluded to by the qualitative research approach. We combined all these questions into one main research question, as is often done in qualitative studies: What is the experience of developing and maintaining resilience in people with CMP?

The next step after formulating the research question was to choose the right research paradigm or perspective on how a researcher sees and interprets the world. In recent studies, resilience has been seen as a context-dependent construct (Gentili et al., 2019; Hayman et al., 2017; Ungar, 2018). Resilience can be understood differently when we discuss, for example, adaptation to chronic pain, the experience of divorce, domestic violence, or childhood trauma. In different contexts, the opportunities for individuals are different, the needs are different, and the extent to which individuals can make use of these opportunities is different (Pooley & Cohen, 2010). Considering that there is no such thing as “common resilience for all”, we decided to ground our research on the paradigm of social constructivism. Constructivists acknowledge that individuals construct their own perceptions of the world, but social constructionists go one step further, arguing that those individual constructions are developed in a social world (Harper & Thomson, 2011). A fundamental assumption of the social constructivism paradigm is that there is no universal reality. Meanings, knowledge, and truth are created by the interactions of individuals within a society (Andrews, 2012; Creswell, 2013).

The choice of the social constructivism paradigm, along with the research question, confirmed the use of a qualitative research strategy, as it is more appropriate to study mental facts, such as experiences, feelings, and attitudes, which are ontologically subjective phenomena. In contrast, a quantitative research strategy is more suitable for studying brute facts or external reality (Silva, 2008). Quantitative studies have made a major contribution to resilience research in healthcare by demonstrating that resilience is positively correlated with social and physical functioning, adaptation to illness and better health outcomes (Kim et al., 2019; Musich et al., 2022; Schäfer et al., 2022; Seiler & Jenewein, 2019), but quantitative studies can’t provide a sufficiently deep and comprehensive understanding of how resilience is formed and how the resilience dynamic is influenced by the general context of life.

Resilience is a multidimensional, contextually speciﬁc, and culturally biased construct (Ungar, 2013). The meaning we put in the words “being resilient” is not the same for all of us. Global resilience is at best quite rare, if not non-existent because it changes in different situations and at different times (Vanderbilt-Adriance & Shaw, 2008). For example, a person can cope effectively with stressors at work but shows very low resilience in the face of disease. These differences can be explained by the fact that resilience is influenced not only by internal but also by external risk and protective factors. Resilience of an individual depends on resilience of interconnected systems. Resilience develops and changes because all of the systems accounting for resilience are dynamic (Masten, 2021). Many authors (Bonanno & Mancini, 2008; Davydov et al., 2010; Geard et al., 2018) admit that resilience in encountering short-term stressors differs from the resilience we experience when living with long-term adversity. Strategies that help in the short term may not be helpful in the long term; besides, we can experience several ups and downs.

Using resilience questionnaires and scales, we can determine some general characteristics or manifestations of resilience. Longitudinal studies allow to measure resilience in different periods of time, but quantitative studies are unable to answer the question of why changes in resilience at different stages of life and in specific situations happen. Qualitative research methods (especially, interviews) could help to understand the meanings, beliefs, and values of the participants, which play a critical role in explaining their behaviour and its consequences and understanding the effect of social and cultural contexts on these meanings, behaviours, processes, and results (Maxwell, 2021).

Although a mixed methods design is often used to study common and unique aspects of resilience (Ungar & Liebenberg, 2011) and initially we considered using the mixed methods research in this study, we came to the conclusion that our research question is related to the deep understanding of participants’ unique experience of resilience and can best be answered by using the qualitative research design. Taking into account the aspects mentioned above, it appears that a qualitative research strategy would be the most appropriate choice to study resilience in people with chronic pain. Furthermore, we have provided arguments for why we have chosen the particular research design.

## Multiple case study design

The case study design was selected as the most relevant to investigate the resilience of people with CMP. Creswell defines a case study as an in-depth exploration of a bounded system or multiple bounded systems in their real-life setting (Creswell et al., 2007). In our research, each case (each participant’s experience of resilience while living with chronic pain) has its limits in time (the duration of the illness) and its unique context or real-life context (environment, available resources, etc.).

In contrast to experimental designs, which seek to test a specific hypothesis through manipulating the environment, the case study approach lends itself well to capturing information on more explanatory questions “how”, “what”, and “why” (Crowe et al., 2011). Case study research is an increasingly popular approach among qualitative researchers, providing methodological flexibility through the incorporation of different paradigmatic positions, study designs, and methods (Hyett et al., 2014).

There are two key approaches to case study research. Those researchers whose philosophical assumptions are grounded in postpositivism usually use Robert Yin’s approach (Yin, 2003), but researchers whose philosophical assumptions are grounded in constructivism mostly use the approach by Robert Stake (1995) or Sharan B. Merriam (2009).

Since we grounded our research in the paradigm of social constructivism, the approach to the case study by Stake was chosen. He emphasizes that a case study is not a methodological choice but rather a choice of what is to be studied (Stake, 2008). In our research, this is the subjective experience of the resilience of each research participant.

Stake and other representatives of constructivism claim that reality is not available to us in an objective way; it is possible to study only the meaning people attach to what has happened because each of us interprets reality differently (Yazan, 2015). In our research, we are not studying resilience as an objective reality that can be measured, but as a subjective perception of this experience over time.

Stake speaks about three types of case studies: intrinsic, instrumental, and multiple case studies (Stake, 1995). An intrinsic case study allows one to explore a unique phenomenon. An instrumental case study is used if a researcher wants to gain a broader understanding of some issue through this particular case, but the collective or multiple case study involves multiple cases being studied simultaneously or sequentially to gain an even broader understanding of the issue. In our study, we apply multiple case design.

Multiple case studies are often used in health psychology (Boblin et al., 2013; Breet & Bantjes, 2017; Fearon et al., 2021), because these studies allow a researcher to analyse within each setting and between settings (Baxter & Jack, 2008). In our investigation, we were interested in individual stories and the unique resilience experience of each participant, but we also wanted to know whether people with chronic pain have used similar strategies to adapt to the disease and if they have mentioned any common factors that helped them develop resilience. In light of the arguments mentioned above, a multiple case study seemed to be the most relevant design.

In the following paragraphs, we will substantiate the selection of specific methods for data collection and analysis and how multi-method and pluralistic approaches can enhance research rigour.

## Multi-method qualitative approach as methodological triangulation

Similarly as in quantitative research, qualitative research has its criteria to ensure the rigour of the research. One such criterion is triangulation. Triangulation means being able to look at the same phenomenon or research topic through more than one source of data (Abdalla et al., 2018). It refers to the use of multiple methods or data sources in qualitative research to develop a comprehensive understanding of phenomena (Patton, 1999). Triangulation is not only a strategy for the validation of the research procedures and results (Flick, 2018) but also a strategy that allows adding depth to the data that are collected and gives a more complete picture of the phenomenon that is studied (Fusch et al., 2018). Abdalla et al. suggest several functions of triangulation. Information from different angles can be used to confirm, develop, or illuminate the research problem (Abdalla et al., 2018).

For more than three decades, qualitative researchers have used multiple forms of triangulation in a study: data triangulation, methodological triangulation, theory or perspective triangulation, and investigator triangulation, following the suggestions of Denzin (Denzin, 1989). By data triangulation, Denzin meant different data points (people, time, space) that represent different data of the same event. By methodological triangulation, he meant multiple data collection methods, for example, interviews, focus groups, and observations. The theory triangulation designated viewing data through the lens of different theories, while the investigator triangulation meant that more than one investigator was observing the same data.

In the study described in this article, we combined two data collection methods that provide methodological triangulation. A combination of several qualitative data collection methods to investigate a research question or phenomenon is usually called the “multiple method(s)” approach (McDonnell et al., 2017) or “multimethod(s)” (Anguera et al., 2018; Mik-Meyer, 2020; Roller & Lavrakas, 2015) approach. Some authors, like Janice M. Morse (Morse, 2003, 2009) have used both concepts. In our research, we use the term “multimethod approach”, which is also used by American Psychological Association (APA, n.d).

The combination of different data-gathering methods allows us to overcome each method’s weaknesses and limitations, contributes to a better understanding of a research problem compared to research that is based on only one methodological approach, and provides knowledge that otherwise is inaccessible to the researcher (Creswell, 2015).

However, some authors admit that multi-method research also has some challenges. One such challenge is how to synthesize the findings of two separate methods if they are not complementary but conflicting (Nepal, 2010). In our study, data gathering methods are complementary, but any contradicting results, if such appear, are analysed assuming that the contradictions may not exist simultaneously but emerge at different time points. In the following paragraphs, we will explain how the combination of several data analysis methods can help to solve these contradictions.

Another challenge is to compare the weight of the data obtained by different methods. For example, does a focus group interview with six participants carry the same weight as an individual interview? (Carter et al., 2014). In addition, this challenge in more detail will be described further.

In our qualitative study, we combine individual semi-structured interviews with focus groups conducted with interviewed participants. In the following sections of this paper, we will explain our considerations for combining these methods and justify why we took both methods onboard with the same participants.

## Combination of semi-structured individual interviews and focus groups

A semi-structured interview (SSI) is the most common format of data collection in qualitative research. It employs a relatively detailed interview guide and is designed to determine subjective responses from people regarding a particular situation or phenomenon they have experienced (McIntosh & Morse, 2015). Although SSI has a pre-planned structure, it differs from a structured interview with more openness. SSI is often accompanied by follow-up “why” or “how” questions (Adams, 2015) and gives the interviewer the opportunity to elaborate and explain particular issues through the use of open questions (Alsaawi, 2014). It also differs from an unstructured interview, where the interviewer asks only some general questions and is mainly a listener (Brinkmann, 2014). SSI is useful when a researcher works with a complex issue because he can use probes and spontaneous questions to explore, deepen understanding, and clarify answers to questions (Wilson, 2014).

We selected SSI as the main data collection method for several reasons: 1) from the main qualitative data collection methods (observations, textual or visual analysis, individual and group interviews) only individual or focus group interviews could give enough information to answer the research question, 2) in a one-to-one interview format, the interviewer can create a safe environment and adjust to every participant; 3) we had a set of specific research subquestions (*How do people with chronic pain describe the development of resilience? How do they describe factors that have contributed to or hindered resilience at the beginning of their illness? How do they describe the manifestation of resilience in the long term? How do they describe factors that have contributed to or hindered resilience in the long term? How does resilience change over time?*), so we needed a fairly structured interview protocol that allowed us to answer these questions. But we also did not want to lose in-depth data and unexpected disclosures, which is why we did not select a structured interview.

Although individual interviews have many advantages, they have some disadvantages as well, such as the hierarchical position and the power of the interviewer over the participant. The participant is reduced to the role of a passive provider of data, while the interviewer is the one who uses skilled rapport promotion technology (Nunkoosing, 2005). Another disadvantage is a lack of group dynamics, which could bring new themes into discussions (Lambert & Loiselle, 2008).

To enhance research rigour, we decided to use one more data collection method and combine individual interviews with focus groups. The focus group approach is a qualitative method for collecting data on the selected topic with a structured and focused discussion in a small group of people (Gundumogula, 2020). Focus groups create open lines of communication between individuals and rely on the dynamic interaction between participants to produce data that would be impossible to gather via other approaches, such as one-on-one interviewing (Jarvis & Barberena, 2008). A significant role in focus groups is played by a moderator. The involvement of a good moderator can ensure that the conversation is always on track and encourage the participation of participants without one individual dominating the discussion (Sagoe, 2012).

For some participants, it could be easier to disclose personal and sensitive information through individual interviews (Kaplowitz, 2000; Kruger et al., 2019), but for others, the focus group format could be more appropriate. Listening to other participants’ experience stories can encourage self-disclosure and stimulate memory (Guest et al., 2017; Kitzinger, 1994).

The limitation of focus groups is the possibility of bias and manipulation through leading or dominating participants, as well as tendencies towards normative discourses, conflicts, and arguments within focus groups (Gundumogula, 2020; Smithson, 2000). Using these methods together, it could be possible to find a balance between looking for a diversity of topics and a deeper investigation of each topic.

Janice Morse argues that in situations where a researcher uses multiple qualitative methods, one of them is usually a core method and the rest methods are supplementary methods. A second qualitative component can identify gaps or holes, “pick up” what the first method missed and allow discussing some parts of the findings that had not been on the researcher’s screen earlier (Morse, 2010).

In our study, a semi-structured interview is a core method that was used to collect data from all participants, while focus group discussions were used as a supplementary method to obtain feedback from the part of research participants who were interviewed and to clarify whether our interpretation of the interview data coincides with the views of the participants. Focus group discussions as a complementary method are also valuable because due to the dynamics of the group, participants could recall important information they did not mention during the interviews. Interaction between participants can promote discussions and bring new perspectives to the investigated problem. Participants can influence each other through their presence and their reactions to what other people say (Mack et al., 2005).

In the first phase of the study, we developed a protocol for the semi-structured interview consistent with the research questions. Because of our decision to use an inductive approach to data analysis, our questions weren’t grounded in the literature and we didn’t have an intention to test hypothesis through the answers to these questions. Instead, we were open to whatever emerged from the data. To avoid the situation where participants could be influenced to give certain answers or very short answers, we formulated only open-ended interview questions aligning with research questions, thus aiming for richer data.

The interviews were approximately 60 to 90 minutes long and provided us with main data on the lived experiences of the participants. Since we were interested in the dynamics of resilience, the interviewer spent a lot of time listening to stories about different periods in the life of the participants. If the participants wanted to share more information than asked, the interviewer allowed them to speak because additional information would help to understand the context of the story and give a deeper understanding of the different factors that have influenced the resilience of the participants.

Our strategy was to analyse the interview data and find out which themes appeared in the participants’ responses more frequently, speaking about each research question. We were also looking for contradictory ideas and trying to understand what influences specific beliefs and values. For example, why do some participants accept the disease as something they will have to live with all their lives, but others still have the hope to eliminate the disease? More information about the data analysis process will be presented in the following chapters of this paper.

After drawing the first conclusions, we organized two focus groups. In the theoretical literature, there is a suggestion to conduct at least two focus groups to ensure data saturation. (Hennink et al., 2019). The more focus groups are organized, the more different themes and perspectives can arise, and the researchers can find ideas that are common in all groups. Since focus groups in our study are only an additional method and the sample is quite small (17 participants), it was agreed that two groups would be enough to get feedback from participants about our interpretations of the research results.

Before moving on to data analysis, we must answer the question of why we stopped collecting data at the point that we did and what our arguments were for determining the sample size.

## Criteria for determining sample size

Samples in qualitative research tend to be small to support the depth of case-oriented analysis, that is fundamental to this mode of inquiry, but at the same time large enough to allow the unfolding of a new and richly textured understanding of the phenomenon under study (Sandelowski, 1996; Vasileiou et al., 2018).

Although qualitative researchers still have discussions about the number of interviews, that would be enough to ensure the research rigour and provide the answers to the research questions, there are several criteria that help to define an optimal sample size. In the thematic analysis, that is used in our research, one of the most significant criteria to determine sample size is saturation. Saturation can be defined as the point at which additional data do not lead to any new emerging themes (Given, 2016). Even if some new codes arise, these data change a little or do not change the coding result at all. According to this criterion, the researcher can stop conducting interviews at the moment when saturation is reached (Bryman, 2012). But this approach, as emphasized by Bryman (2012), is a very demanding one, because it forces the researcher to combine sampling, data collection, and data analysis, rather than treating them as separate stages in a linear process. Another suggestion is that a researcher must be sure that the data he/she has and what he/she wants to say coincide, that data support his/her conclusions, and conclusions are not going beyond what data can support (Becker, 2012).

Hennik et al. acknowledge that saturation can be understood as code saturation and meaning saturation. Code saturation can be defined as the point where no additional issues are identified and the codebook begins to stabilize but meaning saturation can be defined as the point where we fully understand issues and when no further dimensions, nuances, or insights of issues can be found (Hennink et al., 2017). It is easier to reach code saturation than meaning saturation because people can put different meanings in the same codes, and some codes, especially abstract ones, can have multiple dimensions. Focusing on codes alone is a deficient measure of saturation; codes can be saturated, but vital information remains unconsidered (Sebele-Mpofu, 2020). It is important not only to look at the frequency of the data but also to interpret the data and to see what is in it (McIntosh & Morse, 2015).

Saturation is influenced by multiple parameters or criteria that determine how large a sample must be. One such criterion is accessibility. The more specific and harder to access the population, the smaller could be the minimal number of participants (Adler & Adler, 2012; Brannen, 2012). Another criterion is the homogeneity or heterogeneity of the population. In a homogeneous population, the sample size could be smaller; in a heterogeneous population with more different subgroups, the sample must be larger (Adler & Adler, 2012; Brannen, 2012; Hennink et al., 2017). The theoretical background can also influence the sample size (Bryman, 2012; Hennink et al., 2017). For example, life story research is likely to involve a smaller sample size than research aiming to develop some theory. The sample size will most likely be smaller if the data is thick or richer and larger than if the data are thin (Hennink et al., 2017). And, of course, available resources can also play an important role in a sample size (Flick, 2018).

Maltreud and collegues (Malterud et al., 2016) have proposed the concept of “information power” to guide adequate sample size for qualitative studies. Information power depends on the aim of the study, sample specificity, use of established theory, quality of dialogue, and analysis strategy. The more information the sample holds, relevant to the actual study, the lower amount of participants is needed.

By evaluating the criteria mentioned above, we realized that our sample must be rather small, than big, because of quite a narrow and specific aim of our study. The aim of this study is to capture themes, not to develop theories. Although the population under study has subgroups, it is still quite homogeneous. The interviews would produce thick data. The only argument that indicated the need for a larger sample was the multidimensional concept of resilience, which could determine the longer time to move from code saturation to meaning saturation.

In our study, we interviewed 17 people with CMP. To answer our main research question *“What is the experience of developing resilience in people with CMP?”* we purposely looked for working-age participants with different types and different intensities of musculoskeletal pain, such as back pain, joint pain, pain after spinal cord injury, etc., who are 18–65 years old and have been living with pain for five years or more. We approached participants through patient associations, Facebook groups, and personal contacts. There were seven men and ten women among the participants aged 29 to 64 years. Four participants had chronic pain after spinal cord injury and used a wheelchair. Six participants had rheumatoid arthritis or other rheumatoid disease and seven participants had other diagnoses that caused neck or back pain, like spondylosis, osteoporosis, and disk herniation. Three participants didn’t do paid work. Two of them were women at pre-retirement age who looked after their grandchildren and one was a man with a spinal cord injury. The other participants worked despite the limitations caused by pain.

The decision to stop data collection after 17 interviews was based on several considerations. First of all, we reached a code and meaning saturation. In our study, thematic analysis was the instrument to examine saturation. During the first stage of the inductive thematic analysis, we developed a codebook and applied it to the rest of the interviews. Having analysed 13 interviews, we found central codes that are repeated in each interview and that less than 5% of the new codes appear. After we found central codes and reached code saturation, we went through all interviews and analysed what participants mean by each code. Fully understanding all dimensions of conceptual codes requires much more data than fully understanding concrete codes (Hennink et al., 2017). In our study, the category that was described bythe largest diversity of meanings was “adapting to the disease”. For some participants, it meant the ability to handle everything by themselves, but for others—the ability to use available social resources. We continued to conduct interviews and after analysing 17 interviews, we reached meaning saturation because no new code dimensions appeared.

By studying theoretical literature and analysing the criteria mentioned above, we found that sample size, starting from 12 interviews, can be sufficient for data saturation in a thematic analysis (Ando, Cousins, & Young, 2014) and 16 interviews can lead to meaning saturation (Hennink et al., 2017). It matched our conclusion that 17 interviews would give enough information to answer the research question.

After analysing 17 interviews, we obtained sufficient information power, that allowed us to provide a thick description of each case as well as find commonalities and differences between cases.

In the next paragraphs, we will provide more detailed information on the process of data analysis and justify the necessity for a pluralistic approach.

## The pluralistic approach to qualitative data analysis

Previously, we described our assumptions for choosing a qualitative research strategy and considerations for using two data collection methods. In this paragraph, we’ll continue to describe the data analysis process and will demonstrate why the development of resilience as a dynamic process should not be understood as applying only a single method of data analysis.

To describe different aspects of qualitative data, we use the pluralistic data analysis approach. In research, methodology pluralism has been approached using a range of conceptual labels (Frost & Nolas, 2011). In a broader sense, pluralism means combining a range of different data modes in a single research project, for example, quantitative and qualitative methods, but in a more narrow sense, it refers to the combination of several qualitative data analysis methods.

Pluralism in qualitative research is defined as the application of more than one qualitative analytical method to a single data set (Clarke et al., 2014) or, as specified by Frost, as the interpretation of one interview transcript with different qualitative analysis techniques (Frost et al., 2010). The aim of pluralist analyses is to produce rich, multilayered, multiperspective readings of any qualitative data set through the application of diverse ways of seeing and maximizing holistic understanding (Dewe & Coyle, 2014).

According to the literature, multiple analytical approaches are appropriate for understanding a plural and complex world, and the variety of human expression cannot always be adequately represented by one framework alone (Chamberlain et al., 2011; Frost et al., 2010; Kincheloe, 2001, 2001). The data set can tell us several different things, depending on the questions we ask. Analysing the same data from different analytical lenses can reveal more meanings than analysing these data just from one analytical lens (Frost et al., 2010; Willig, 2013). The pluralistic approach not only enhances a deeper understanding of the phenomenon but, if each analysis method is performed by different researchers, it also reduces subjectivity and increases transparency in a study (Frost et al., 2010).

The pluralistic approach is widely used in social sciences; in recent years, it has also gained popularity in health psychology research (Dempsey et al., 2019; Dewe & Coyle, 2014; Madill et al., 2018; Rosas et al., 2019). The pluralistic approach has several advantages but combining different data analysis methods can also be challenging.

Researchers must find ways to demonstrate coherent links between theory, method, and findings and explain how findings produced from multiple analyses can remain commensurate or complementary (Braun & Clarke, 2019; Clarke et al., 2014). There must be a clear rationale for the theories and methods being used so that the researchers demonstrate reflexivity and document their research process in an accessible manner (Frost & Nolas, 2011). The use of methods without justification can lead to disjointed and fragmented findings (Chamberlain et al., 2011). Another challenge is the willingness of researchers to use a pluralistic approach. Pluralism requires researchers to be competent in all methods they apply (Clarke et al., 2014), which could be challenging, especially for new researchers.

In our study, we investigate both the content and dynamics of the experience of resilience in people living with chronic pain. Therefore, we are interested not only in resilience development strategies and factors that positively or negatively influence resilience but also in changes over time—how these strategies and factors change if we compare short-term and long-term resilience.

Upon starting this research, our main focus was on strategies that help to improve resilience. We considered that thematic analysis could be the best data analysis method for finding the most common strategies. After conducting the first pilot interviews, we were surprised by the richness of the available data. The participants shared different stories of their lives and acknowledged that the way they perceive pain has changed over time. We realized that we must broaden our research question and focus not only on common themes but also on the life of each participant in its unique context and dynamic. Therefore, we decided to apply both thematic and narrative analysis to analyse our data. Then, after conducting the third pilot interview, we noticed an interesting nuance—all participants were speaking about specific turning points in their lives, which dramatically changed their attitudes and resilience. From this, we understood that we need one more method that could be appropriate for analysing those changes. Studying the literature, we found that the critical incident technique (CIT) could be valuable to define critical incidents or experiences that contributed, positively or negatively, to resilience.

The pluralistic approach was not our strategy at the beginning of the investigation, but we came to this decision during the analysis of the pilot interviews. It confirms once again that conducting pilot interviews is an especially important step that allows for identifying “holes” and flaws in research questions and methods. The combination of thematic analysis, narrative analysis, and critical incident technique could provide answers to all research questions that we are interested in. More detailed considerations of the use of each method will be illustrated in the next paragraphs.

## Combining methods: thematic analysis, narrative analysis, and critical incident technique

At the beginning of the research, our focus was mainly on strategies that help improve resilience. We decided that a thematic analysis would be an appropriate method to find common themes and to find out which strategies to improve resilience would be the most helpful. The interview protocol was created, and three pilot interviews were conducted and analysed with reflexive thematic analysis approach created by Braun and Clarke (Braun & Clarke, 2006). Pilot studies allow researchers to practice and assess the effectiveness of their planned data collection and analysis techniques (Doody & Doody, 2015). The piloting of interviews was set up to find out whether the interview questions are understandable and provide answers to the research question.

After conducting and analysing three pilot interviews, we realized that qualitative data provide more comprehensive material than we initially expected. We observed that interviewees not only answered the questions but spoke about their life as a whole, bringing up significant experiences from their past, like other traumatic experiences (such as divorce or losing their job), important people in their lives that influenced their values and attitudes, the brightest childhood memories, etc.

We concluded that we must revise the interview protocol and, for further interviews, include more questions about the dynamics of experience in different stages of the disease. This was the first time we noticed that short-term and long-term strategies differ, so the questions should be modified from more general to more specific. Creswell et al. (2007) has emphasized that qualitative research questions could change during the entire research process. Initial provisional questions can become more focused because researchers gain a deeper or broader understanding. That is why the qualitative study could not be fully planned in advance.

Since we added new research questions, we also needed new methods for data analysis. We realized that it is impossible to answer all research questions by using only thematic analysis. The thematic analysis allows one to find common themes between cases (Braun & Clarke, 2006; Joffe, 2012) but the narrative analysis could be more appropriate for analysing differences in cases and describing the dynamics of individual narratives in their unique context (Floersch et al., 2010; Simons et al., 2008).

Pilot interviews gave us rich qualitative data, including information about events that dramatically changed participants’ attitudes and resilience. So, we concluded that in addition to thematic and narrative analysis, CIT could be valuable for defining critical incidents or experiences that made a contribution, either positively or negatively, to resilience. Finally, we decided to combine reflexive thematic analysis (Braun & Clarke, 2006), narrative analysis (Crossley, 2000), and the enhanced critical incident technique (ECIT) (Butterfield et al., 2009). In what follows, the use of each method is explained in detail.

### Reflexive thematic analysis

Thematic analysis (TA) can be seen as an umbrella term, used for sometimes quite different approaches, rather than a single qualitative analytic approach. The three main approaches in TA are the coding reliability approach, the codebook approach, and the reflexive approach (Braun & Clarke, 2019). TA has been widely used in recent qualitative health research designs (e.g., Lyng et al., 2022; Opsomer et al., 2019; Zarotti et al., 2019), because it is not strictly connected with a particular methodology and is quite flexible.

Since our research is based on the paradigm of social constructivism, we decided to use a reflexive thematic analysis. An interpretive or social constructivist approach to qualitative case study research supports a transactional method of inquiry, where the researcher has a personal interaction with the case (Hyett et al., 2014). Of all TA approaches, reflexive TA fits best with the paradigm of social constructivism because it emphasizes the active role of the researcher in coding and theme generation. The researcher not only identifies semantic themes and summarizes the content of the data, but also looks for latent themes, revealing the underlying ideas within the data (Braun & Clarke, 2019). The subjectivity of a researcher is the primary “tool” for reflexive TA. Subjectivity is not a problem to be managed or controlled, it is a resource for research (Braun & Clarke, 2019; Gough & Madill, 2012 as cited by).

The described investigation focuses not on objective reality but on the way participants perceive and, together with the researcher, interpret their subjective experiences. It should also be acknowledged that the previous experiences, biases, and research position of researchers impact the way they look at the data. Subjectivity without reflexivity could be a limitation, but if researchers are aware of their role and impact, subjectivity could become a resource. In this study, the researcher, who conducted the interviews, is an insider to the study population. The researcher’s personal experience of living with chronic pain helped stimulate a dialogue with interviewees and increase mutual trust.

In recent years reflexive TA has been used more often in health psychology (Bose & L Elfström, 2022; D’Souza et al., 2022; McKenna-Plumley et al., 2021), since it is a theoretically flexible method and could be adapted to different research designs.

By using a classic six-step process (Braun & Clarke, 2006): 1) familiarizing oneself with the data, 2) generating codes, 3) constructing themes, 4) reviewing potential themes, 5) defining and naming themes, and 6) producing the report, we gradually moved through the data several times until we constructed final themes. The thematic analysis allowed us to answer “what” questions about the content of resilience. What strategies do people with chronic pain use to promote resilience? What are the main obstacles and contributing factors?

### Narrative analysis

After identifying central themes with TA, we assumed the narrative analysis of each case. Just like thematic analysis, narrative analysis is an umbrella term, not a single method. The narrative method allows us to look at the story from a holistic perspective without the need of breaking it down into themes (Riessman, 2008). Narrative not only brings order and meaning to our daily life but, reflexively, it also provides structure to our very sense of self-hood (Murray, 2015). The narrative analysis helped us answer questions that start with “how”, for example, how people see the impact of disease on their lives and how they describe changes in their habits, attitudes, and life as a whole while living with chronic pain.

We based our analysis on the Michelle Crossley’s (2000) framework that includes six steps:

1) reading and familiarizing, 2) identifying important concepts to look for, 3) identifying “narrative tone”, 4) identifying the “imagery” and themes, 5) weaving it all together, and 6) writing a research report.

Since we study resilience in the context of chronic pain, the Crossley’s framework seemed to be the most appropriate one, as the author has developed this framework to analyse stories of illness and trauma. In health psychology, the Crossley’s framework is frequently used (Manning, 2015; Winslow et al., 2005; Wong & Breheny, 2021). Crossley has admitted that when people talk or write about their experiences of chronic or serious illness, they often characterize themselves as becoming totally different people (Crossley, 2000). Resilience often means not just bouncing back or returning to a status quo but bouncing forward or becoming even stronger than before illness (Hynes et al., 2020). This change could also be perceived as becoming a totally different person. In our research, we were interested in this process of change. The narrative analysis allowed us to answer “how” questions about the resilience process. How does disease change our attitudes towards ourselves and others? How does time influence these changes?

We applied narrative analysis for each research question in each interview and analysed responses for different stages of the disease. For example, asking about strategies people used to overcome or accept pain, we looked at what the strategies were and how they changed in the first months after diagnosis, in the first years after diagnosis, and in the long term, five or more years after diagnosis. This timeline provided an opportunity to study the dynamics of resilience. The creation of an approximate timeline helped to understand why particular themes appear in the specific moment after diagnosis and how they are related to other life events.

### The critical incident technique

Finally, we applied CIT to qualitative data to describe the ups and downs that significantly changed people’s lives. The founder of CIT is John Flanagan (1954), who developed this method for the Aviation psychology program of the US army. The purpose of the CIT was to gather information on behaviours that contribute to the success or, in contrast, lead to failure.

Flanagan’s technique was rooted in the positivist paradigm and was more suitable for studying job performance in the field of organizational psychology. After more than 50 years Lee D. Butterfield and colleagues (Butterfield et al., 2009) modified this method so that it could meet the needs of researchers from multiple perspectives and could be used in different fields, and named this method ECIT.

In our research, we apply the ECIT which is methodologically more flexible than Flanagan’s technique and could be adjusted to the paradigm of social constructivism. ECIT allows us to study critical incidents from the perspective of the participants and explore their perception of the main turning points, without the expectation that we are studying the objective reality. Compared to other methods, ECIT is a relatively rarely used method in qualitative research, but several recent studies prove that this method could be a good research tool in psychology (Klarare et al., 2018; Kwee et al., 2020; Nitkin & Buchanan, 2020; Springer & Bedi, 2021).

ECIT involves five main steps: 1) determining the general goals of the activity being studied, 2) making plans and setting specifications, 3) collecting the data, 4) analysing the data, and 5) interpreting the data and reporting the results. Although the main steps are defined very generally, Butterfield describes in detail how to perform each step. For example, he illustrates how to identify critical incidents (something that helped or hindered a particular experience or activity) and wish list items (those people, support, information, programs, etc., that were not present at the time of the participant’s experience, but those involved believe would have been helpful) (Butterfield et al., 2009).

To ensure credibility and rigour, Butterfield also developed nine credibility checks for ECIT—audiotaping interviews, interview fidelity, independent extraction of critical incidents, exhaustiveness, participation rates, placing incidents into categories by an independent judge, cross-checking by participants, expert opinions, and theoretical agreement (Butterfield et al., 2009).

When analysing critical incidents, we also looked at the approximate timeline to find out whether critical incidents were related to the time since diagnosis.

To conclude, we can say that all three methods allowed us to answer different research questions, complement each other, and help achieve the research objectives (see Table I). In the next chapter, we will describe how we integrated all three data analysis methods and how the within-case and across—case approach helped to achieve a balance between generalization and an in-depth understanding of the particular case.

**Table I.** Research questions and data analysis methods.

Research Question	Data Analysis Method
How do people with chronic musculoskeletal pain (CMP) describe the development of resilience?	Thematic analysis allows one to identify topics that people talk about when recalling the development of resilience. Narrative analysis allows one to describe the dynamics of the experience.
How do people with CMP describe the factors that have contributed or hindered resilience?	Thematic analysis allows one to identify what the main obstacles and contributing factors are that people talk about. Narrative analysis allows one to understand which factors dominate in different stages of illness.
How do people with CMP describe changes in resilience over time?	Thematic analysis allows us to identify the main changes that people talk about. Narrative analysis allows one to describe the dynamics of the experience or the sequence of change. It allows us to understand how these changes are related to other life events. The critical incident technique allows one to answer the question: Which internal processes or external events have been a turning point that significantly changed people’s lives?
How do people with CMP describe the long-term manifestation of resilience?	Thematic analysis allows one to identify the topics that people talk about and find common long-term strategies. Narrative analysis allows one to describe the dynamics of the experience or the way in which short-term solutions become long-term solutions.

## Within-case and across-case approach in the data analysis process

Case study research has sometimes been criticized for lacking scientific rigour and providing little basis for generalization (Crowe et al., 2011; Hammersley et al., 2000; Kyburz-Graber, 2004). Although Stake (1995) argues that the purpose of case study research is particularization, not a generalization, the goal of researchers who are doing multiple case research is not only an in-depth understanding of particular cases but willingness to provide findings that could be applied to other similar contexts.

Considering that generalizability due to a small sample size could be a problem, qualitative researchers instead speak about qualitative generalization or transferability as one of the trustworthiness criteria (Anney, 2014; Levitt, 2021; Maxwell, 2021). Qualitative generalization or transferability means that findings are described in a thick way or in such detail that readers can see both constancy and variation within a phenomenon and transfer data from the study to their own context (Levitt, 2021). The researcher must provide enough information on the meanings, contexts, and processes operating in the study setting or population that the reader can adequately judge (Maxwell, 2021).

To ensure that findings are reported widely and transparently enough, in the beginning, the researcher should create a system of how he/she will integrate all data analysis methods and notice common elements in a rich material of data, gathered from individual cases.

In our research, we applied within-case and across-case analysis, described by Lyoness Ayres et al. (Ayres et al., 2003) as an approach that helps to achieve qualitative generalization and find a balance between uniqueness and differences from one side and commonalities from the other. Across-case analysis means looking for common themes in all accounts, within-case analysis means in-depth exploration of a single account, considering contextual richness. In multiple case studies, integration of across-case, and within-case analysis is often used (Banerjee & Dixit, 2016; Chung, 2019; Fearon et al., 2021; Glette & Wiig, 2022; Starks et al., 2010), because it allows producing contextually grounded, generalizable findings (Ayres et al., 2003).

Within-case methods are less useful in the development of generalizations about the experience of health and disease drawn from multiple cases, but they provide contextual richness. Neither across-case nor within-case approaches alone enable the researcher to interpret an experience both through its parts and as a whole so that readers can recognize individual experiences in a generalizable way (Ayres et al., 2003).

For example, if we look only at cases and analyse common themes, we could find several controversial themes, such as denial of the disease and acceptance of the disease. But if we look at the cases and each person’s story as a whole, we can see that in the first months after diagnosis the person can deny the disease and avoid talking about health problems, but after a while, the disease could become part of his daily life.

The within-case and across-case approach also allows for the investigation of situations where most of the cases have similarities, but some cases differ from others. Looking across and within cases, we can identify possible factors that could influence these differences (past experience, social factors, thinking patterns, religiosity, etc.). For example, if we analyse the acceptance process, we can see that most patients have accepted their condition, but in some cases, the participants do not accept the fact that they will have to live with this diagnosis for the rest of their lives. By examining these diverse cases in more detail, we can see that these people believe in God’s healing.

By combining the within-case and across-case approach, we could find a balance between generalization and an in-depth understanding of the experience of resilience while living with chronic pain.

## Conclusions

The purpose of this paper was to describe the decision-making chain of a qualitative research process and, specifically, to discuss how the integration of several methods of data collection and analysis can improve a deeper understanding of the formation and maintenance of resilience among people with CMP.

Although qualitative researchers have many methodological freedoms, sometimes this freedom can become a pitfall. If a researcher lacks tacit knowledge of different approaches and their theoretical basis, he/she may choose methods that are inconsistent with each other or inappropriate for answering the research questions. In this paper, we provide an example of how to avoid these pitfalls. We briefly describe each step we were doing and provide transparency for the readers so that they can follow the analysis process.

At the beginning, we formulated the research question: *What is the experience of developing and maintaining resilience in people with chronic musculoskeletal pain (CMP)?*

Considering that resilience can be understood differently in different contexts and that we can explore only subjective interpretations of resilience, but not resilience as such, we decided to ground our research on the paradigm of social constructivism. A fundamental assumption of the social constructivism paradigm is that meanings, knowledge, and truth are created by the interactions of individuals within a society.

When we had chosen the paradigm or perspective of how we will look at the experience of resilience, we decided to use a qualitative research strategy that is more appropriate for studying subjective constructs, such as experiences, feelings, and attitudes at different stages of life and in specific situations. This article approves that the qualitative research strategy can provide a significant contribution to health psychology. It allows analysing of complex constructs and helps not only to identify the problem but also to reveal the causality and influence of various factors on the situation.

The next step was to choose a research design. Since we were interested not only in the unique resilience experience of each participant but also wanted to know if people with chronic pain have used similar strategies to adapt to the disease, we concluded that multiple case study designs will allow us to analyse within each setting and between settings.

In this paper, we have provided arguments on how a multimethod approach can promote research rigour. We combined two data collection methods, semi-structured interviews and focus groups. Semi-structured interviews gave us rich material of data and allowed us to answer concrete subquestions but focus group discussions were a supplementary method for getting feedback from participants and clarifying our interpretations.

We also described the process of determining the sample size. The decision to stop data collection after 17 interviews were based on several considerations. We got enough information to answer the research question and reached code and meaning saturation.

The data analysis process is the most time-consuming part of qualitative research, especially if researchers have chosen a pluralistic data analysis approach and interpreted an interview transcript with different qualitative analysis techniques. In this paper, we argue why it is worth doing it. Analysing the same data from different analytical lenses can enhance a deeper understanding of the construct, reveal more meanings, and give a holistic understanding compared to analysing these data from only one analytical lens.

It is very important to conduct pilot interviews to see if the chosen data analysis method can provide answers to the research questions. At the beginning of our research, we considered that in our study thematic analysis could be the best data analysis method to find the most common strategies. However, after conducting the first pilot interviews, we were surprised by how rich the data was. Participants shared the dynamics of their experience while living with chronic pain, as well as information about events that dramatically changed their attitudes and resilience. We came to the conclusion that we must revise the interview protocol and include more questions and additional data analysis methods.

Finally, we decided to combine three methods, thematic analysis, narrative analysis, and CIT. The thematic analysis allowed us to find common themes between cases, narrative analysis was more appropriate for analysing differences in cases and describing the dynamics of individual narratives in their unique context, while the critical incident technique was valuable for defining critical incidents or experiences that made a contribution, either positively or negatively, to resilience.

To find a balance between uniqueness and differences, on the one hand, and commonalities, on the other hand, we applied within-case and across-case approach in the data analysis process. This allowed us to explain controversial topics and identify possible factors that could influence differences between cases, as well as give contextual richness.

The decision-making chain described in this article can serve as an example for qualitative researchers interested in health research, especially those who study lived experiences of resilience or other constructs in its dynamics and unique context, like dynamics of health behaviour, changes in professional health, self-regulation in the context of chronic diseases etc.

It’s important to justify and make transparent every decision during the process of qualitative research not only because it increases the quality of the research in the eyes of other researchers, but also because it helps to convince policymakers and stakeholders that qualitative research just like quantitative research could be well-grounded and can give a significant contribution to society. To engage in dialogue with decision-makers and wider society, findings should be presented in an easily understandable way by putting an emphasis on practical solutions this research can promote. The strength of this paper is the strong connection between theory and practice. Examples of specific studies can be helpful to better understand the theoretical assumptions and recommendations. The limitation of this study is the small sample size and heterogeneity of participants who have different kinds of musculoskeletal pain, such as back pain, joint pain, or spastic pain. For further studies, it would be valuable to analyse the results in different subgroups of participants to see whether strategies to improve resilience differ depending on the severity of the disease and the type of pain.

## Ethical approval

This study was approved by the Riga Stradiņš University Research Ethics Committee.
